# A feasibility study of returning clinically actionable somatic genomic alterations identified in a research laboratory

**DOI:** 10.18632/oncotarget.16018

**Published:** 2017-03-08

**Authors:** Natalia Paez Arango, Lauren Brusco, Kenna R. Mills Shaw, Ken Chen, Agda Karina Eterovic, Vijaykumar Holla, Amber Johnson, Beate Litzenburger, Yekaterina B. Khotskaya, Nora Sanchez, Ann Bailey, Xiaofeng Zheng, Chacha Horombe, Scott Kopetz, Carol J. Farhangfar, Mark Routbort, Russell Broaddus, Elmer V. Bernstam, John Mendelsohn, Gordon B. Mills, Funda Meric-Bernstam

**Affiliations:** ^1^ Department of Surgical Oncology, The University of Texas MD Anderson Cancer Center, Houston, TX, USA; ^2^ Sheikh Khalifa Bin Zayed Al Nahyan Institute for Personalized Cancer Therapy, The University of Texas MD Anderson Cancer Center, Houston, TX, USA; ^3^ Department of Bioinformatics and Computational Biology, The University of Texas MD Anderson Cancer Center, Houston, TX, USA; ^4^ Department of Systems Biology, The University of Texas MD Anderson Cancer Center, Houston, TX, USA; ^5^ Gastrointestinal Medical Oncology, The University of Texas MD Anderson Cancer Center, Houston, TX, USA; ^6^ Levine Cancer Institute, Carolinas Healthcare System, Charlotte, NC, USA; ^7^ Department of Hematopathology, The University of Texas MD Anderson Cancer Center, Houston, TX, USA; ^8^ Department of Pathology, The University of Texas MD Anderson Cancer Center, Houston, TX, USA; ^9^ School of Biomedical Informatics, The University of Texas Health Science Center at Houston, TX, USA; ^10^ Division of General Internal Medicine, Medical School, The University of Texas Health Science Center at Houston, TX, USA; ^11^ Department of Genomic Medicine, The University of Texas MD Anderson Cancer Center, Houston, TX, USA; ^12^ Department of Investigational Cancer Therapeutics, The University of Texas MD Anderson Cancer Center, Houston, TX, USA

**Keywords:** precision medicine, genomics, somatic mutation, CLIA, clinical trial

## Abstract

**Purpose:**

Molecular profiling performed in the research setting usually does not benefit the patients that donate their tissues. Through a prospective protocol, we sought to determine the feasibility and utility of performing broad genomic testing in the research laboratory for discovery, and the utility of giving treating physicians access to research data, with the option of validating actionable alterations in the CLIA environment.

**Experimental design:**

1200 patients with advanced cancer underwent characterization of their tumors with high depth hybrid capture sequencing of 201 genes in the research setting. Tumors were also tested in the CLIA laboratory, with a standardized hotspot mutation analysis on an 11, 46 or 50 gene platform.

**Results:**

527 patients (44%) had at least one likely somatic mutation detected in an actionable gene using hotspot testing. With the 201 gene panel, 945 patients (79%) had at least one alteration in a potentially actionable gene that was undetected with the more limited CLIA panel testing. Sixty-four genomic alterations identified on the research panel were subsequently tested using an orthogonal CLIA assay. Of 16 mutations tested in the CLIA environment, 12 (75%) were confirmed. Twenty-five (52%) of 48 copy number alterations were confirmed. Nine (26.5%) of 34 patients with confirmed results received genotype-matched therapy. Seven of these patients were enrolled onto genotype-matched targeted therapy trials.

**Conclusion:**

Expanded cancer gene sequencing identifies more actionable genomic alterations. The option of CLIA validating research results can provide alternative targets for personalized cancer therapy.

## INTRODUCTION

There is increasing interest in genomic profiling to personalize cancer therapy. However, very few genomic tests are routinely used as biomarkers for molecularly targeted treatment selection, such as human epidermal growth factor receptor 2 (HER2) for breast cancer or testing for *KRAS* in colorectal cancer and *BRAF* for melanoma. The “Single gene, single test approach” [1] misses multiple alterations that could be targeted in clinical trials with novel therapeutics and others that may represent markers of resistance to standard therapies. While next-generation sequencing (NGS) has revolutionized the detection of clinically actionable alterations by testing multiple genes simultaneously in a relatively cost-effective, tissue-sparing manner, [2] this technology is still not widely accessible in many Clinical Laboratory Improvement Amendments (CLIA) certified laboratories.

There has been a rapid evolution of molecular testing, with multiple new techniques becoming widely available in research laboratories, such as targeted panel exome sequencing, whole-exome sequencing, and RNAseq, among others. There is often a long lag from incorporating new technologies from the research setting to implementation into the CLIA laboratory. This lag is, in large part, due to the fact that many molecular tests are not yet considered “standard of care,” and thus not implemented in the CLIA environment, as they are not billable and payable. Testing for research purposes is usually performed in a non-CLIA-certified environment and this data is not accessible to treating physicians or patients, even if actionable alterations are discovered. And even though there is already growing awareness of the potential to identify incidental germline findings and experience with returning these results to patients [3], there have been no systematic attempts to make results from somatic tests done in the research setting with newer technologies accessible to treating physicians, and to facilitate validation and return of relevant results to patients to guide patient care.

Due to the growing physician and patient demand for genomic profiling, and the inability to utilize results obtained in the research setting to aid in clinical decision-making, we initiated a prospective clinical study where physicians were able to enroll patients who they felt would benefit from multiplex genomic testing, and where patients were likely to be considered for enrollment in therapeutic clinical trials. 1200 patients underwent genomic testing on a Clinical Laboratory Improvement Amendments (CLIA) compliant platform after informed consent, with subsequent high depth hybrid capture sequencing in the non-CLIA research laboratory for discovery. The protocol allowed for treating physicians to have access to the research data through a specific interface. Here, we report the frequency of identifying additional alterations in these 1200 patients, the feasibility of CLIA validating research results, and the utility of this approach as determined by physician utilization of this additional genomic information and subsequent patient enrollment onto clinical trials.

## RESULTS

### Detection of somatic alterations

The tumors of 1200 patients with different types of advanced cancer were tested in the CLIA laboratory with a standardized hotspot mutation analysis on an 11, 46 or 50 gene platform. Five hundred twenty-seven (44%) of 1200 patients had at least one somatic mutation detected in an actionable gene, with a total of 663 mutations in 30 genes. Using the deep targeted sequencing platform (T200) under the research setting, 686 of the 1200 patients (57%) had a total of 2448 previously undetected mutations in potentially clinically actionable genes (Table 2). The deep coverage across exons in the research platform allows sensitive detection of mutations with MAF as low as 1% as described in Chen et al. [4]. Six hundred forty-three (97%) of 663 mutations detected in the CLIA setting were also reported with the research platform. (eTable 2 in the supplement). In addition 12 mutations (2%) were concordant but below the reporting threshold, 2 mutations (0.3%) were indels that were not being reported on the research platform at the time the informatics analysis was done. Six mutations (1%) were discordant. As seen in Table 2, the likelihood of identifying hotspot mutations, as well as additional alterations, differed by disease.

**Table 1:** Patient demographics

**Age, years**		
	Median	53
	Range	2 - 84
**Sex**		No. Patients (%)
	Female	694 (58)
	Male	506 (42)
**Ethnic origin**		
	Asian	40 (3)
	Black or African American	75 (6)
	Hispanic	112 (9)
	White	935 (78)
	Other	34 (3)
	NR	4 (<1)
**Tumor types**		
	Brain	198 (17)
	Breast	281 (23)
	GI	224 (19)
	GYN	62 (5)
	Skin	165 (14)
	Sarcoma	132 (11)
	Other	138 (12)

Abbreviations: GI, Gastrointestinal; GYN, Gynecological; NR, not recorded

**Table 2:** Number of patients with alterations in potentially actionable genes

	All Patients	Patients with mutations detected in CLIA hotspot limited gene panel	Patients with previously unknown mutations in deep targeted sequencing platform	Patients with potential copy number alterations in deep targeted sequencing platform
Tumor type	No. (%) n=1200	n=527	n=686	n=654
Brain	198 (17)	101 (51)	125 (63)	108 (55)
Breast	281 (23)	89 (32)	152 (54)	185 (66)
GI	224 (19)	136 (61)	135 (60)	103 (46)
GYN	62 (5)	18 (29)	19 (31)	36 (58.1)
Skin	165 (14)	128 (78)	127 (77)	87 (53)
Sarcoma	132 (11)	17(13)	49 (37)	69 (52)
Other	138 (12)	38 (28)	79 (57)	66 (48)

Abbreviations: GI, Gastrointestinal; GYN, Gynecological

Unlike hotspot analysis, high depth hybrid capture sequencing also allows detection of copy number variations, and we found that 654 (55%) of 1200 patients had a total of 2784 copy number alterations in 69 potentially actionable genes, including both amplifications and deletions that had been previously undetected (Figure 2). New alterations were detected in 945/1200 patients (79%). Research testing detected 5232 alterations not detected on CLIA testing (Figure 3A).

**Figure 1:**
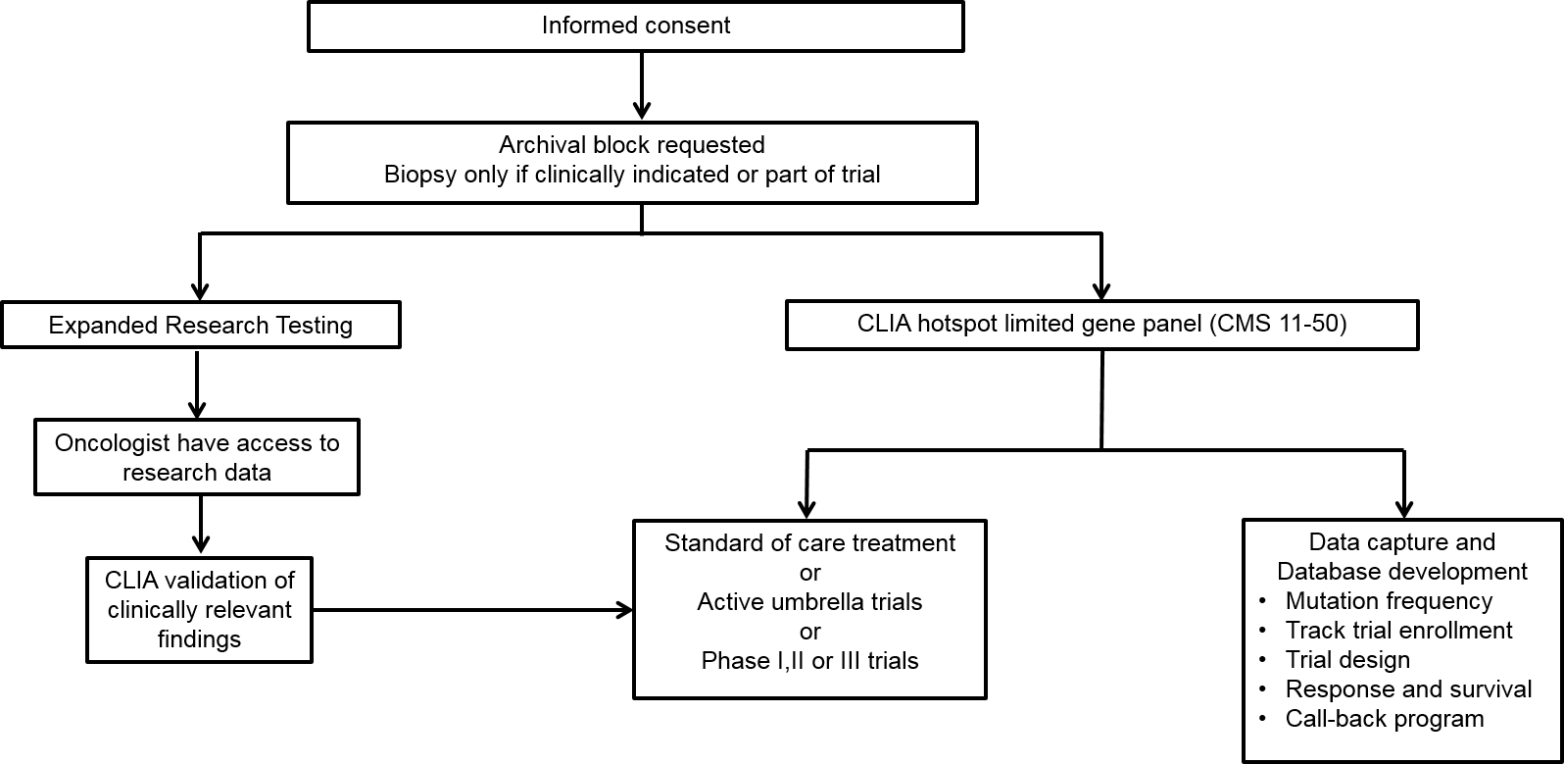
Study design for identification of clinically actionable somatic genomic alterations

**Figure 2:**
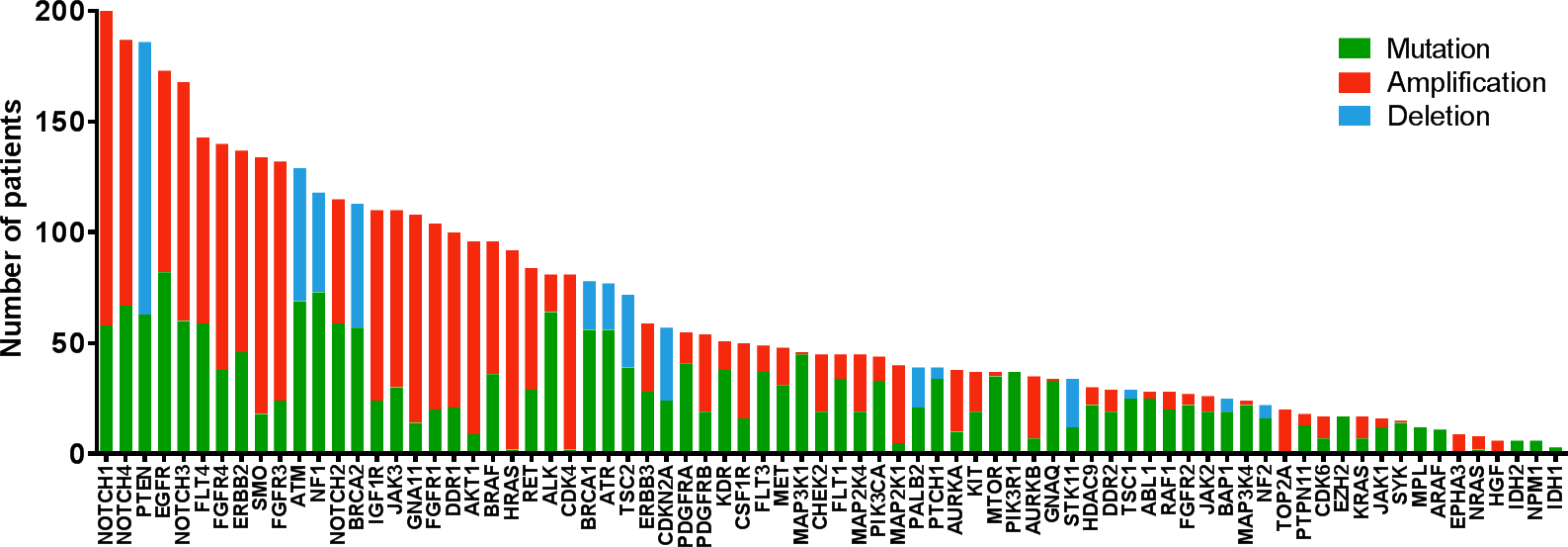
Number of patients with previously unknown actionable mutations (green), Amplification (red) and Deletions (blue) for each gene detected in the T200 platform

**Figure 3:**
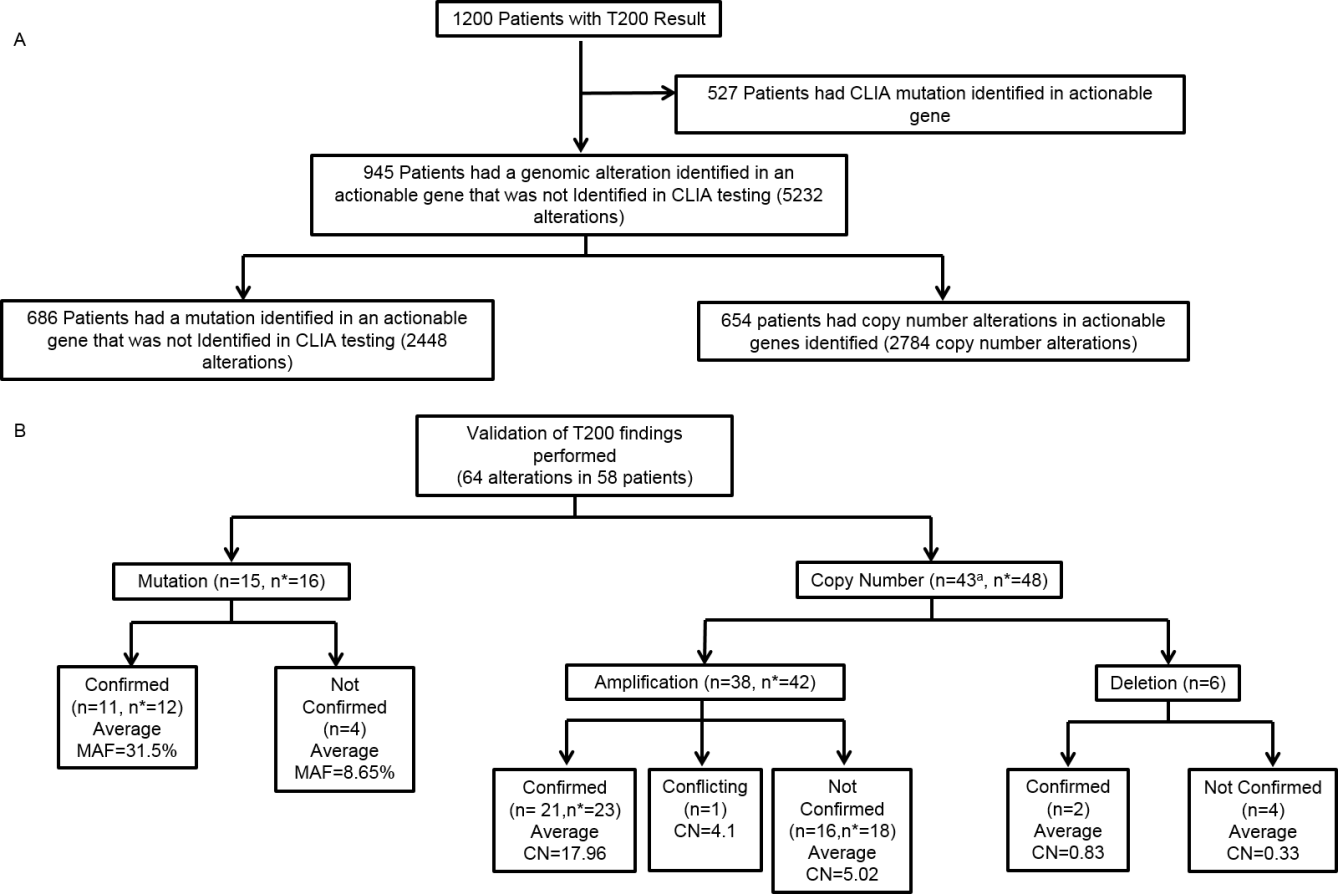
Detection and validation of somatic alterations using hybrid capture sequencing in research setting **A**. Somatic alterations detected using hybrid capture sequencing. *n* = number of patients. **B**. CLIA validation of T200 findings. *n* = number of patients, n* = number of alterations, ^a^ = Overall numbers remove duplication of patients that had both amplification and deletion concomitantly. MAF = Mutant allelic frequency, CN = Copy number.

### Turnaround time for research testing

Time from consent to obtaining the CLIA result was a median of 25 days (range 7-466 days), with much of this time being spent locating archival tissue samples. As expected, timelines for research testing were much longer, since the tissue was released for research only after CLIA testing. Median time from consent to obtaining T200 results was 220 days (range 77-783 days). This decreased the potential clinical utility of findings in the research laboratory since in this patient population with advanced disease, 239 of 1200 patients (20%) were deceased by the time research testing results were available. However, we need to keep in mind that the primary endpoint of the research testing was not to drive clinical care, thus the timeline was not originally designed to directly impact clinical outcomes and any research findings that were CLIA validated represented new potential treatment options that would have otherwise not been identified.

### Physician engagement

The treating physician was notified with an email alert when results of the T200 tests were available. The 1200 patients were distributed among 101 physicians with the mean of 12 patients per physician (range 1-55 patients per physician). Of these, 43 physicians (43%) accessed the results for a total of 471 of 1200 patients (39%). Physicians that accessed research results, accessed the records of a median of 90% of their own patients (range 1-100%). Twenty-six (26%) physicians reviewed over 80% of their patients’ research records. Physicians that accrued more 5 patients or more on the study were more likely to access the research data (*p* = 0.0029; eFigure 1 in the Supplement).

It should be noted that all physicians agreed to pursue CLIA confirmation of any new findings on the T200 platform prior to initiating any treatment based on the research results, and were able to access research results only after acknowledging this expectation. Further, at the IRB request, physicians were not allowed to share research data with their patients without CLIA validation. During the duration of our study, to our knowledge, only one physician inadvertently shared a research finding with a patient prior to CLIA validation. This finding was indeed validated in a CLIA lab shortly thereafter and the patient did not receive any therapy based on this research finding. This event was reported to the IRB and collaborating physicians were further educated.

### CLIA validation

Using an orthogonal CLIA assay, 64 alterations were sent for validation in 58 patients. These 58 patients were selected based as they were deemed to be potential candidates for clinical trials based on their clinical and performance status. Of those, 16 were mutations found in 15 patients. Twelve (75%) of these 16 mutations were confirmed (Table 3). The patients in whom the mutations were confirmed had a higher mutant allelic frequency (MAF; median 30.7% vs. 8.7%; *p* = 0.005). Indeed, 11 of 12 mutations with a MAF of 10% or higher were confirmed.

**Table 3:** Number of alterations sent for validation

	Mutations			Copy number alterations		
Gene	n=16	Confirmed No. (%) n=12	Average MAF	n=48	Confirmed No. (%) n=25	Average Copy Number
ALK	1	1 (100)	30	0	0 (n/a)	N/A
AKT1	0	0 (n/a)	N/A	2	0 (0)	4
ATM	1	1 (100)	39	0	0 (n/a)	N/A
CDK4	0	0 (n/a)	N/A	3	3 (100)	17
CDKN2A	2	2 (100)	24	0	0 (n/a)	N/A
EGFR	1	0 (0)	9	10	10 (100)	24.4
ERBB2	0	0 (n/a)	N/A	15	4 (27)	7
FGFR1	1	1 (100)	12	4	4 (100)	11.7
FGFR3	1	0 (0)	8	3	0 (0)	6
Met	0	0 (n/a)	N/A	5	2 (40)	8
MTOR	1	1 (100)	30	0	0 (n/a)	N/A
NF1	1	0 (0)	8	0	0 (n/a)	N/A
PIK3R1	3	2 (67)	17	0	0 (n/a)	N/A
PTEN	4	4 (100)	44	6	2 (33)	0.7
	16	12 (75)		48	25 (52)	

N/A: Not Applicable, MAF= Mutant allelic frequency

Of the 64 alterations sent for validation (on a number of platforms as outlined in eTable 3 in the Supplement), 48 were copy number alterations found in 43 patients. Twenty-five (52%) of those 43 alterations were confirmed and 18 were not confirmed (Figure 3B). One was a *MET* amplification that was confirmed with IHC, but showed no amplification on FISH. Copy number alterations that were not confirmed had an average estimated copy number of 5, compared to 18 for confirmed alterations (eTable 3 in the Supplement). Six copy number deletions were detected and selected for validation (by IHC), all in the *PTEN* gene: 2 of these patients were found to have PTEN loss by IHC.

### Clinical utilization of high depth hybrid capture sequencing findings

Nine patients with new alterations detected by T200 testing and that were validated with the appropriate orthogonal CLIA test subsequently received genotype-matched therapy. Seven of these enrolled onto clinical trials for targeted therapy based on their genomic alteration. It is important to note that this is a markedly higher percentage than for patients found to have mutations on original hotspot CLIA testing [5]. The alterations targeted included two patients with glioblastoma that had *MET* amplifications, one patient with melanoma with *CDK4* amplification, a breast cancer patient with an *FGFR1* amplification, a patient with colorectal cancer with an *ALK* mutation, a colorectal cancer patient with a *PIK3R1* mutation, and a leiomyosarcoma patient with a *PTEN* mutation. Of the 26 patients that were not enrolled in trials, 14 (54%) were not enrolled due to poor performance status.

Four patients were found to have HER2 amplification. One breast cancer patient who was previously diagnosed with HER2 negative disease on CLIA testing with IHC was found to have HER2 amplification on research testing. This patient received HER2-targeted therapy based on the new biomarker subtyping. One patient with salivary cancer received HER2-targeted therapy off-label. Two additional patients with HER2 amplification were offered clinical trial participation: one with colon cancer, and the other with bladder cancer. In both cases, the investigator-initiated trials required off-label targeted therapy coverage, which was declined by insurance.

## DISCUSSION

While large-scale genomic testing to facilitate enrollment onto clinical trials is feasible, it remains challenging [5]. Large-scale testing is not readily available in many centers, and with “hotspot” genotyping or targeted sequencing with limited gene panels, the majority of patients do not have actionable alterations detected [6]. Expanding the number of genes tested and incorporating copy number testing can identify additional patients with alterations in potentially actionable genes. This can provide treatment options to individual patients through clinical trials and track the frequency and associated outcomes of specific alterations in each disease across multiple histologies, giving insights into the functional relevance of these alterations by helping discern which are “drivers” versus “passengers” [1, 7]. High depth hybrid capture sequencing facilitated the detection of 5232 previously unrecognized alterations, detecting at least one additional alteration in 79% of the patients tested. As the technology for cancer sequencing continues to evolve, the research efforts are exponentially growing to develop newer and better platforms, and while patients generously donate their tissues to help this cause, it is frustrating that we are not able to routinely act upon the results found in the research setting. For this reason we want to show that we are potentially able to utilize these additional results that are found in the research setting for clinical decision making by having a prospective framework that allows CLIA validation and therefore benefit patients by giving them more treatment options.

Overall, patients and physicians are very interested in clinical genomic testing, as demonstrated by the rapid accrual to this study. However, physicians varied in whether they accessed the non-CLIA research data through the cancer genome mutation browser. There were high volume physicians who accrued several dozen patients, but did not access research (non-CLIA) results, while some other high volume accruers accessed each of their patient's research records. Notably, in a prior study in another institution, over a fifth of physicians surveyed expressed low confidence in their genomic knowledge [8]. In our study, there was a significant association between the number of patients physicians accrued to the study and whether or not they accessed the research data (*p* = 0.0029). It is likely that faculty that were high accruers and those that accessed research data felt more comfortable with genomics or were more interested in offering genomically-informed therapy, and thus these physicians represent “early adopters” of genomics.

Given the high cost of genomic testing, doing high-throughput somatic testing in the research environment with CLIA validation of actionable alterations is one approach that has been considered. However, even finding archival samples in a timely manner and doing CLIA testing is challenging. In patients with progressive advanced disease, sequentially doing research testing and CLIA validation is less likely to facilitate next line treatment selection. Thus, if this approach were to be adopted, methodologies to initiate research testing earlier in treatment course would be needed. However, given the large amount of molecular profiling that is already occurring in academic centers, it is important that we consider novel approaches to derive maximal benefit from that testing, including return of actionable results from research testing to the physician and patient after CLIA validation when there is potential clinical utility. Notably, we and others have already embarked on efforts to return incidental pathogenic germline findings [3].

In previous work, we found that 98.2% of mutations identified by CLIA 46 gene testing were also identified by T200 [4]. Further, to estimate the false discovery rate of T200, we randomly selected 98 novel T200 mutations that were not covered by the AmpliSeq46 platform, not found in the COSMIC v63, and had allele frequencies >10%. When we retested them using Sequenom MassArray™, 96 of 98 mutations were validated, suggesting a 2.0% false discovery rate [4]. In this report, 75% of the mutations were validated in an orthogonal CLIA test. Alterations that were not validated occurred at a lower allelic frequency (mean 8.7% vs. 30.7%). It is possible that such alterations are at a level below the sensitivity of the CLIA test that was used; further, understanding of the limit of detection of the various assays is needed [7]. However, the 11 of 12 mutations at 10% or higher MAF were validated. One could argue that mutations that are of lower MAF may not be truncal mutations and are less compelling targets, and thus we were able to confirm the most compelling actionable alterations. As CLIA samples for validation were different samples (but usually from same biospecimen), tumor heterogeneity may also be a contributor to cases that were not confirmed. Further, we used a variety of different platforms for validation, including assessing PTEN IHC to assess PTEN loss, etc., thus it is possible that this further decreased validation rate.

Due to the clinical importance of HER2, we attempted to validate borderline cases (eTable 3), as well as cases predicted to be highly amplified, which likely contributed to our low confirmation rate. Using FISH, we confirmed HER2 amplification in four patients, and in all four HER2-targeted therapy was offered. In two of the four patients who were offered HER2-targeted therapy were treated with HER2-targeted therapy. Two additional patients with HER2 amplification were offered clinical trial participation: one with colon, and the other with bladder cancer. In both cases, the investigator-initiated trials required off-label targeted therapy coverage, which was declined by insurance. Unfortunately, after both patients were deceased, a multicenter HER2-selected basket trial was activated and demonstrated activity in both diseases [9].

Seven patients were enrolled onto targeted therapy trials based on genomic alterations in *ALK*, *CDK4*, *MET*, *FGFR1*, *PTEN*, and *PIK3R1* detected on the T200 platform followed by validation. Although only a small number of patients ultimately enrolled on trials, this likely reflects challenges with using data that was delayed by months from the original sequencing request. Further, it is important to note that the percentage of patients with research tests subsequently confirmed by CLIA who went on genomically driven trials was higher than the percentage of patients where mutations are found on the original CLIA testing who went on genomically driven trials in the same period [5]. And while optimally, in order to be able to fully impact patient care, the initial test a patient undergoes would ideally be an expanded genomic test in the CLIA setting where physicians can act upon the results in a timely matter as the data is available, the possibility to be able to use data obtained from the research setting to further impact patient care represents an important value added. Further, the ability to return research test represents for a platform for discovery and research-driven patient care, independent of research testing approach used. We are currently expanding our research testing in the Clearinghouse study to testing that will include DNA, RNA, and proteomic analysis. Taking into account that the low number of physicians accessing the research data may signify discomfort with the genomics we have now, a prospective decision support team, with results being reviewed on a clinical trial alert basis has been implemented to also help with variant interpretation and identifying therapeutic implications upon clinical testing. We hope that the framework we developed to facilitate return of actionable alterations will expand treatment options for future patients.

## MATERIALS AND METHODS

### Patient selection and enrollment

From March 2012 to June 2014, 4407 patients with documented invasive cancer and likely to benefit from somatic genomic testing were enrolled into an Institutional Review Board-approved protocol: “Molecular Testing for the MD Anderson Cancer Center Personalized Cancer Therapy Program” (NCT01772771) also known as the “clearinghouse protocol” and underwent CLIA hotspot genomic testing. From this cohort 1200 patients had adequate amount of tissue left for further research testing, and after the CLIA sequencing was successfully completed, testing was followed by high depth hybrid capture sequencing of the tumor in the research environment (Figure 1). All patients had metastatic or advanced disease and the demographics are outlined in Table 1.

### Genomic analysis

First-line testing for characterization of tumors was performed with high throughput genotyping of common genomic alterations in the CLIA environment of MD Anderson Cancer Center Molecular Diagnostic Laboratory. Testing was done on archival diagnostic tissue on an 11 gene Sequenom MassArray SNP genotyping platform or on a 46 or 50 gene Ion Torrent AmpliSeq platform as previously reported [5, 10] (Supplementary methods in the supplement). If adequate residual DNA was available, an aliquot of the DNA extracted in the CLIA setting was used for more extensive analysis of somatic mutations in the research setting by high depth hybrid capture sequencing (T200) as previously described [4]. If residual DNA from the original extraction was inadequate, DNA was extracted in the research laboratory from additional slides sectioned at the same time as the initial CLIA testing. Slides were reviewed by pathologists at the MDACC tissue qualification laboratory and areas of tumor cellularity were identified for macro-dissection to enhance tumor cellularity for DNA extraction in the CLIA environment. Subsequent slides were macro-dissected for research testing.

### Access to research data and CLIA validation

Treating physicians were given the opportunity to access the research data on their patients through an internal secure portal, the Cancer Genome Mutation Browser (CGMB) after agreeing not to act on the results without CLIA validation. Data obtained in the research lab was not placed in the patient record. Physicians were sent an email notification when research data became available. In addition to providing access to research data, the portal allowed physicians to request validation of relevant results in a CLIA compliant laboratory. Notably, this aspect of the protocol was quite controversial, and led to multiple reviews by the MD Anderson IRB, including the executive IRB, and legal team. Ultimately, it was felt that the risk to patients in this study was outweighed by the potential benefits, and the protocol was approved.

We defined actionable genes as those that are therapeutically actionable based on their potential to be targeted with approved or investigational therapies [11, 12]. At the time of our analysis we considered a total of 80 potentially actionable genes in the T200 platform (eTable 1 in the Supplement).

Physicians were given the opportunity to request CLIA validation of potentially actionable research findings if it was felt that CLIA validation would increase therapeutic options for the patient. In addition, the MD Anderson Precision Oncology Decision Support (PODS) team regularly reviewed the results for clinical actionability based on available trials for most of the patients, taking into account patient performance status and eligibility, as described by Johnson et. al [12], and discussed potential role of clinical validation with treating physicians.

Mutations identified by the T200 platform were validated in the CLIA environment with Sanger sequencing or PCR with custom-generated probes. Gene amplifications were CLIA validated with fluorescent in situ hybridization (FISH), comparative genomic hybridization, molecular probe inversion arrays, or CLIA next-generation sequencing (based on availability) to assess copy number, or with immunohistochemistry (IHC) to assess protein overexpression. *PTEN* gene deletions were validated by demonstrating loss of PTEN protein expression by IHC as previously described [13].

## SUPPLEMENTARY MATERIALS FIGURES AND TABLES




