# Correction: A feasibility study of returning clinically actionable somatic genomic alterations identified in a research laboratory

**DOI:** 10.18632/oncotarget.27176

**Published:** 2019-08-27

**Authors:** Natalia Paez Arango, Lauren Brusco, Kenna R. Mills Shaw, Ken Chen, Agda Karina Eterovic, Vijaykumar Holla, Amber Johnson, Beate Litzenburger, Yekaterina B. Khotskaya, Nora Sanchez, Ann Bailey, Xiaofeng Zheng, Chacha Horombe, Scott Kopetz, Carol J. Farhangfar, Mark Routbort, Russell Broaddus, Elmer V. Bernstam, John Mendelsohn, Gordon B. Mills, Funda Meric-Bernstam

**Affiliations:** ^1^ Department of Surgical Oncology, The University of Texas MD Anderson Cancer Center, Houston, TX, USA; ^2^ Sheikh Khalifa Bin Zayed Al Nahyan Institute for Personalized Cancer Therapy, The University of Texas MD Anderson Cancer Center, Houston, TX, USA; ^3^ Department of Bioinformatics and Computational Biology, The University of Texas MD Anderson Cancer Center, Houston, TX, USA; ^4^ Department of Systems Biology, The University of Texas MD Anderson Cancer Center, Houston, TX, USA; ^5^ Gastrointestinal Medical Oncology, The University of Texas MD Anderson Cancer Center, Houston, TX, USA; ^6^ Levine Cancer Institute, Carolinas Healthcare System, Charlotte, NC, USA; ^7^ Department of Hematopathology, The University of Texas MD Anderson Cancer Center, Houston, TX, USA; ^8^ Department of Pathology, The University of Texas MD Anderson Cancer Center, Houston, TX, USA; ^9^ School of Biomedical Informatics, The University of Texas Health Science Center at Houston, TX, USA; ^10^ Division of General Internal Medicine, Medical School, The University of Texas Health Science Center at Houston, TX, USA; ^11^ Department of Genomic Medicine, The University of Texas MD Anderson Cancer Center, Houston, TX, USA; ^12^ Department of Investigational Cancer Therapeutics, The University of Texas MD Anderson Cancer Center, Houston, TX, USA

**This article has been corrected:** The Acknowledgments information has been updated. The proper Acknowledgments section is shown below:

## ACKNOWLEDGMENTS

This work was supported in part by NCI 5T32CA009599, CPRIT RP 150535, the Sheikh Khalifa Al Nahyan Ben Zayed Institute for Personalized Cancer Therapy, NCI U01 CA180964, NCATS grant UL1 TR000371 (Center for Clinical and Translational Sciences), the Nellie B. Connally Breast Cancer Research Endowment, and the MD Anderson Cancer Center Support Grant (CCSG) (P30 CA016672) and the CCSG supported Sequencing and Microarray Facility (SMF).

Original article: Oncotarget. 2017; 8:41806–41814. 41806-41814 . https://doi.org/10.18632/oncotarget.16018

