# Supplementary material for: A feasibility study of returning clinically actionable somatic genomic alterations identified in a research laboratory
Source: Oncotarget. 2017 Mar 8;8(26):41806–14. doi: 10.18632/oncotarget.16018 (PMC5522029; doi:10.18632/oncotarget.16018)
Supplement: Supplementary file 1 [file oncotarget-08-41806-s001.pdf]

A feasibility study of returning clinically actionable somatic genomic alterations identified in a research laboratory

Supplementary Material

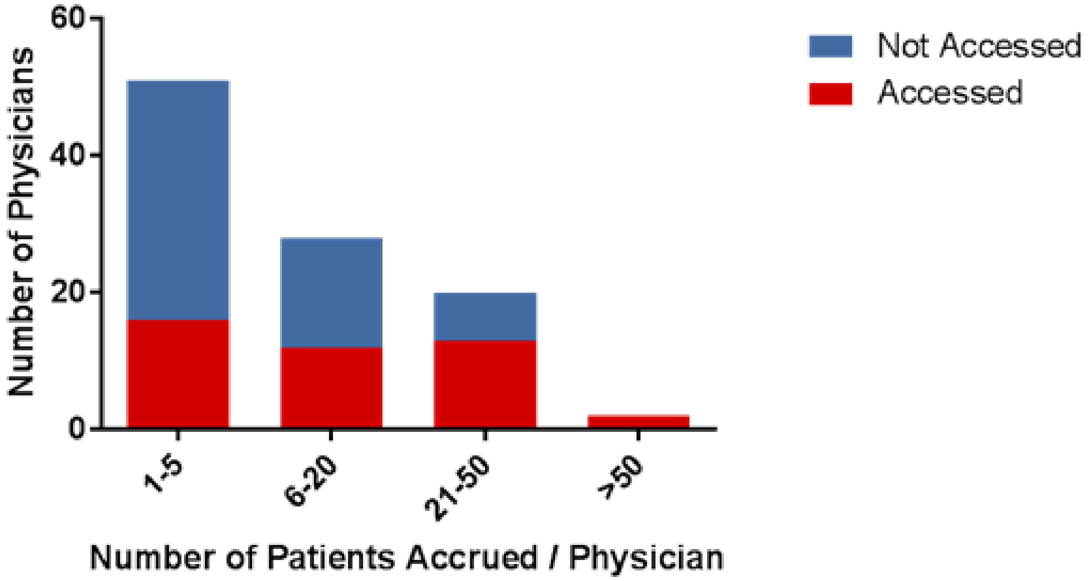

eFigure 1: Number of patients accrued to the study per physician. Number of physicians that accessed the research test results of their patients by how many patients they accrued to the study.

eTable 1. List of genes targeted in T200 platform

eTable 2. List of concordance between the CLIA and research platform

eTable 3. Copy number alterations sent for validation

For e Tables 1,2,3 see supplementary Information

Supplementary methods

Processing of Specimens and Storage of Residual DNA

Fresh frozen tissue samples where stored until processed at -80C. DNA extraction from FFPE or fresh frozen material done in the CLIA environment in the Department of Pathology for CLIA testing. Extraction for research studies was done either at the Institute of Personalized Cancer Therapy laboratory, or in the Meric-Bernstam lab, or through MD Anderson's CCSG Biospecimen Extraction facility.
The method of extraction Depend on the nature of the sample: if there was a low percentage of tumor the samples were to be microdissected prior to extraction.
For extraction of cfDNA from plasma or serum, Qiagen's QiaAMP circulating nucleic acid kit was used. The istitutional guidelines for the maximum allowable amount of blood which can be drawn at one time from patients according to their age was followed.

Molecular Analysis

CLIA testing was performed on on an 11 gene Sequenom MassArray SNP genotyping platform or on a 46 or 50 gene Ion Torrent AmpliSeq platform. The IT AmpliSeq cancer panel is a multiplex polymerase chain reaction–based library preparation method that interrogates 190 regions comprising 740 mutational hotspots in the coding sequence of 46 cancer-related genes. The platform performs DNA sequencing by synthesis and detects the release of hydrogen ions during the incorporation of nucleotides during strand synthesis. The change in pH is detected by an ion-sensitive field effect transistor and converted into sequence information by a signal-processing software. A mutant allele frequency of 10% was required to call a mutation in the patient sample as further described in Roy-Chowdhuri, S., et al., *Multigene clinical mutational profiling of breast carcinoma using next-generation sequencing*
