# Supplementary material for: A feasibility study of returning clinically actionable somatic genomic alterations identified in a research laboratory
Source: Oncotarget. 2017 Mar 8;8(26):41806–14. doi: 10.18632/oncotarget.16018 (PMC5522029; doi:10.18632/oncotarget.16018)
Supplement: Supplementary file 2 [file oncotarget-08-41806-s002.docx]

| **eTable1. List of genes targeted in T200 platform** | |  |
| --- | --- | --- |
| **Gene** | **Potentially Actionable Alterations** | **Potential Therapeutic Implications** |
| ABL1 | Activating mutations, gene amplification, or BCR-ABL1 fusion | Treatment with ABL or BCR-ABL inhibitors |
| ACVR1B | Not considered actionable at time of analysis |  |
| ADAMTS12 | Not considered actionable at time of analysis |  |
| AKAP3 | Not considered actionable at time of analysis |  |
| AKT1 | Activating mutations or gene amplification | Treatment with AKT or mTOR inhibitors |
| ALK | Activating mutations, gene amplification, or ALKgene fusions | Treatment with ALK inhibitors |
| APC | Not considered actionable at time of analysis |  |
| AR | Not considered actionable at time of analysis |  |
| ARAF | Activating mutations | Treatment with RAF inhibitor |
| ARID1A | Not considered actionable at time of analysis |  |
| ASXL1 | Not considered actionable at time of analysis |  |
| ATM | Inactivating mutations or deletions | Treatment with PARP inhibitors |
| ATR | Inactivating mutations or deletions | Treatment with PARP inhibitors |
| AURKA | Activating mutations or gene amplification | Treatment with AURKA inhibitors |
| AURKB | Activating mutations or gene amplification | Treatment with AURKB inhibitors |
| ATRX | Not considered actionable at time of analysis |  |
| BAI3 | Not considered actionable at time of analysis |  |
| BAP1 | Inactivating mutations or deletions | Treatment with HDAC inhibitors |
| BRAF | Activating V600 mutations | Treatment with BRAF/ MEK/ERK inhibitors |
|  | Activating non-V600 Mutations | Treament with MEK and ERK inhibitors |
|  | Intermediate or Inactivating mutations | Treatment with pan-RAF or ERK Inhibitors |
|  | gene amplification | Treatment with MEK, ERK or pan-RAF inhibitors |
|  | Fusions | Treatment with MEK or pan-RAF inhibitors |
| BRCA1 | Inactivating mutations or deletions | Treatment with PARP inhibitors |
| BRCA2 | Inactivating mutations or deletions | Treatment with PARP inhibitors |
| CARD11 | Not considered actionable at time of analysis |  |
| CASP8 | Not considered actionable at time of analysis |  |
| CBL | Not considered actionable at time of analysis |  |
| CD19 | Not considered actionable at time of analysis |  |
| CDH1 | Not considered actionable at time of analysis |  |
| **Gene** | **Potentially Actionable Alterations** | **Potential Therapeutic Implications** |
| CDH10 | Not considered actionable at time of analysis |  |
| CDH11 | Not considered actionable at time of analysis |  |
| CDK4 | Activating mutations or gene amplification | Treatment with CDK 4/6 inhibitors |
| CDK6 | Activating mutations or gene amplification | Treatment with CDK 4/6 inhibitors |
| CDKN2A | Inactivating mutations or deletions | Treatment with CDK 4/6 inhibitors |
| CEBPA | Not considered actionable at time of analysis |  |
| CHEK1 | Not considered actionable at time of analysis |  |
| CHEK2 | Activating mutations or gene amplification | Treatment with Chk2 inhibitor |
| COL14A1 | Not considered actionable at time of analysis |  |
| CPAMD8 | Not considered actionable at time of analysis |  |
| CREBBP | Not considered actionable at time of analysis |  |
| CRIPAK | Not considered actionable at time of analysis |  |
| CSF1R | Activating mutations or gene amplification | Treament with CSF1R monoclonal antibody and inhibitors |
| CSMD1 | Not considered actionable at time of analysis |  |
| CSMD2 | Not considered actionable at time of analysis |  |
| CSMD3 | Not considered actionable at time of analysis |  |
| CTNNB1 | Not considered actionable at time of analysis |  |
| CYLD | Not considered actionable at time of analysis |  |
| CYP2C19 | Not considered actionable at time of analysis |  |
| DAXX | Not considered actionable at time of analysis |  |
| DDR1 | Activating mutations or gene amplification | Treatment with DDR1 inhibitor |
| DDR2 | Activating mutations or gene amplification | Treatment with DDR2 inhibitor |
| DNMT3A | Mutations | High risk" factor of myeoldysplastic or myeloproliferative disorders required for trial enrollment. |
| EGFR | Activating mutations or gene amplification | Treatment with EGFR inhibitors |
| ELN | Not considered actionable at time of analysis |  |
| EML4 | Not considered actionable at time of analysis |  |
|  |  |  |
| **Gene** | **Potentially Actionable Alterations** | **Potential Therapeutic Implications** |
| EP300 | Not considered actionable at time of analysis |  |
| EPHA3 | Amplification | Treatment with Dasatinib |
| ERBB2 | Activating mutations or gene amplification | Treatment with HER2 inhibitors, monoclonal antibodies, and targeted vaccines |
| ERBB3 | Activating mutations or gene amplification | Treatment with HER3 inhibitors |
| ERCC3 | Not considered actionable at time of analysis |  |
| ERCC4 | Not considered actionable at time of analysis |  |
| ERCC5 | Not considered actionable at time of analysis |  |
| ETV5 | Not considered actionable at time of analysis |  |
| EZH2 | Mutations | Treatment with EZH2 inhibitors |
| FAM123B | Not considered actionable at time of analysis |  |
| FAM135B | Not considered actionable at time of analysis |  |
| FAT3 | Not considered actionable at time of analysis |  |
| FBXW7 | Not considered actionable at time of analysis |  |
| FGFR1 | Activating mutations, gene amplification, or gene fusions | Treatment with FGFR1 inhibitors |
| FGFR2 | Activating mutations, gene amplification, or gene fusions | Treatment with FGFR2 inhibitors |
| FGFR3 | Activating mutations, gene amplification, or gene fusions | Treatment with FGFR3 inhibitors |
| FGFR4 | Activating mutations or gene amplification | Treatment with FGFR4 inhibitors |
| FLG | Not considered actionable at time of analysis |  |
| FLT1 | Activating mutations or gene amplification | Treatment with FLT1 inhibitors |
| FLT3 | Activating mutations, gene amplification, or gene fusions | Treatment with FLT3 inhibitors |
| FLT4 | Activating mutations or gene amplification | Treatment with FLT4 inhibitors |
| FOXL2 | Not considered actionable at time of analysis |  |
| GABRA6 | Not considered actionable at time of analysis |  |
| GABRB3 | Not considered actionable at time of analysis |  |
| GATA1 | Not considered actionable at time of analysis |  |
| GATA3 | Not considered actionable at time of analysis |  |
| GNA11 | Activating mutations or gene amplification | Treatment with PKC and MEK inhibitors |
| **Gene** | **Potentially Actionable Alterations** | **Potential Therapeutic Implications** |
| GNAQ | Activating mutations or gene amplification | Treatment with PKC and MEK inhibitors |
| GNAS | Not considered actionable at time of analysis |  |
| HDAC9 | Activating mutations or gene amplification | Treatment with HDAC9 inhibitors |
| HEATR7B2 | Not considered actionable at time of analysis |  |
| HGF | Gene amplification | Treatment HGF monoclonal antibody |
| HMCN1 | Not considered actionable at time of analysis |  |
| HNF1A | Not considered actionable at time of analysis |  |
| HNF1B | Not considered actionable at time of analysis |  |
| HRAS | Activating mutations or gene amplification | Treatment with MEK inhibitors |
| HYDIN | Not considered actionable at time of analysis |  |
| IDH1 | Activating mutations | Treatment with IDH1 inhibitors |
| IDH2 | Activating mutations | Treatment with IDH2 inhibitors |
| IGF1R | Activating mutations or gene amplification | Treatment with IGF1R monoclonal antibodies or inhibitors |
| IKZF1 | Not considered actionable at time of analysis |  |
| IL6R | Not considered actionable at time of analysis |  |
| IRS1 | Not considered actionable at time of analysis |  |
| ITGA4 | Not considered actionable at time of analysis |  |
| JAK1 | Activating mutations or gene amplification | Treatment with JAK inhibitors |
| JAK2 | Activating mutations or gene amplification | Treatment with JAK inhibitors |
| JAK3 | Activating mutations or gene amplification | Treatment with JAK inhibitors |
| KCNB2 | Not considered actionable at time of analysis |  |
| KDM6A | Not considered actionable at time of analysis |  |
| KDR | Activating mutations or gene amplification | Treatment with KDR inhibitors |
| KIT | Activating mutations or gene amplification | Treatment with KIT inhibitors |
| KRAS | Activating mutations, gene amplification, or gene fusions | Treatment with MEK Inhibitors |
| LAMA1 | Not considered actionable at time of analysis |  |
| LPHN3 | Not considered actionable at time of analysis |  |
|  |  |  |
| **Gene** | **Potentially Actionable Alterations** | **Potential Therapeutic Implications** |
| LRP1 | Not considered actionable at time of analysis |  |
| LRP1B | Not considered actionable at time of analysis |  |
| LRP2 | Not considered actionable at time of analysis |  |
| MAP2K1 | Activating mutations or gene amplification | Treatment with MEK Inhibitors |
| MAP2K4 | Activating mutations or gene amplification | Treatment with JNK1 inhibitor |
| MAP3K1 | Activating mutations or gene amplification | Treatment with JNK1 inhibitor |
| MAP3K4 | Activating mutations or gene amplification | Treatment with JNK1 inhibitor |
| MDN1 | Not considered actionable at time of analysis |  |
| MECOM | Not considered actionable at time of analysis |  |
| MEN1 | Not considered actionable at time of analysis |  |
| MET | Activating mutations, gene amplification, or gene fusion | Treatment with MET inhibitors (Crizotinib, Cabozantinib) |
| MITF | Not considered actionable at time of analysis |  |
| MLH1 | Not considered actionable at time of analysis |  |
| MLL2 | Not considered actionable at time of analysis |  |
| MLL3 | Not considered actionable at time of analysis |  |
| MPL | Activating mutations | Treatment with JAK2 inhibitors. |
| MSH2 | Not considered actionable at time of analysis |  |
| MSH6 | Not considered actionable at time of analysis |  |
| MTOR | Activating mutations or gene amplification | Treatment with mTOR inhibitors |
|  | Selected binding domain muations | Resistance to rapalogs |
| MYD88 | Not considered actionable at time of analysis |  |
| NAV3 | Not considered actionable at time of analysis |  |
| NCOR1 | Not considered actionable at time of analysis |  |
| NF1 | Inactivating mutations or deletions | Treatment with PI3K pathway inhibitors (PI3K/AKT/MTOR), MAPK pathway inhibitors (RAF/MEK/ERK), or HSP90 inhibitors |
| NF2 | Inactivating mutations or deletions | Treatment with PI3K pathway inhibitors (PI3K/AKT/MTOR), MAPK pathway inhibitors (RAF/MEK/ERK), HSP90 inhibitors, or FAK inhibitors |
| NFKB2 | Not considered actionable at time of analysis |  |
| **Gene** | **Potentially Actionable Alterations** | **Potential Therapeutic Implications** |
| NOTCH1 | Activating mutations, gene amplification, or gene fusion | Treatment with Gamma Secretase inhibitors (GSIs) |
| NOTCH2 | Activating mutations or gene amplification | Treatment with GSIs |
|  | Gene fusion | Resistance to GSIs |
| NOTCH3 | Activating mutations or gene amplification, or gene fusion | Treatment with GSIs |
| NOTCH4 | Activating mutations or gene amplification | Treatment with GSIs |
| NPM1 | Mutations | Correlate with positive response to all-trans retinoic acid therapy and chemotherapy in AML. |
| NRAS | Activating mutations or gene amplification | Treatment with MEK inhibitors |
| NSD1 | Not considered actionable at time of analysis |  |
| PALB2 | Mutations or homozygous deletion | Treatment with PARP inhibitors |
| PAPPA2 | Not considered actionable at time of analysis |  |
| PAX5 | Not considered actionable at time of analysis |  |
| PBRM1 | Not considered actionable at time of analysis |  |
| PCDH15 | Not considered actionable at time of analysis |  |
| PCLO | Not considered actionable at time of analysis |  |
| PDGFRA | Activating mutations, gene amplification, or gene fusions | Treatment with PDGFRA inhibitors |
| PDGFRB | Activating mutations, gene amplification, or gene fusions | Treatment with PDGFRB inhibitors |
| PIK3CA | Activating mutations or gene amplification | Treatment with PI3K, AKT, or mTOR inhibitors |
| PIK3CG | Activating mutations or gene amplification | Treatment with PIK3CB inhibitors |
| PIK3R1 | Inactivating mutations | Treatment with PI3K, AKT or mTOR inhibitors |
|  |  | Trial selecting for mutations |
| PIKFYVE | Not considered actionable at time of analysis |  |
| PKHD1 | Not considered actionable at time of analysis |  |
| PKHD1L1 | Not considered actionable at time of analysis |  |
| PPP1R3A | Not considered actionable at time of analysis |  |
| PPP2R1A | Not considered actionable at time of analysis |  |
| PPP2R4 | Not considered actionable at time of analysis |  |
| PRDM1 | Not considered actionable at time of analysis |  |
| PRSS1 | Not considered actionable at time of analysis |  |
| **Gene** | **Potentially Actionable Alterations** | **Potential Therapeutic Implications** |
| PTCH1 | Inactivating mutations or deletions | Treatment with SMO inhibitors |
| PTEN | Inactivating mutations or deletions | Treatment with p110beta, AKT, or mTOR inhibitors |
| PTK2 | Not considered actionable at time of analysis |  |
| PTPN11 | Activating mutations or gene amplification | Treatment with MEK Inhibitors |
| RAD51 | Not considered actionable at time of analysis |  |
| RAF1 | Gene amplification | Potential resistance to RAF inhibitors |
|  | Gene amplification, activating mutations, or gene fusions | Treatment with MEK inhibitors |
|  | Deletion or loss-of-function mutations | Resistance to Dasatinib |
| RB1 | Not considered actionable at time of analysis |  |
| RELN | Not considered actionable at time of analysis |  |
| RET | Activating mutations, gene amplification, or gene fusions | Treatment with Ret inhibitors |
| RIMS2 | Not considered actionable at time of analysis |  |
| RNF213 | Not considered actionable at time of analysis |  |
| RUNX1 | Gene fusions | Trials selecting for RUNX1 gene fusions. Treatment with AML1(RUNX1)-ETO chemical inhibitors, cotricosteroids, and methylprednisolone. |
| RUNX1T1 | Not considered actionable at time of analysis |  |
| RYR2 | Not considered actionable at time of analysis |  |
| SETD2 | Not considered actionable at time of analysis |  |
| SMAD4 | Not considered actionable at time of analysis |  |
| SMARCA4 | Not considered actionable at time of analysis |  |
| SMARCB1 | Not considered actionable at time of analysis |  |
| SMO | Activating mutations or gene amplification | Treatment with SMO inhibitors |
| SOS1 | Not considered actionable at time of analysis |  |
| SPEN | Not considered actionable at time of analysis |  |
| SPOP | Not considered actionable at time of analysis |  |
| SPTA1 | Not considered actionable at time of analysis |  |
| STK11 | Inactivating mutations or deletions | Treatment with mTOR inhibitors or AMPK activators |
| **Gene** | **Potentially Actionable Alterations** | **Potential Therapeutic Implications** |
| SYK | Activating mutations or gene amplification | Treatment with Syk inhibitors |
| SYNE1 | Not considered actionable at time of analysis |  |
| SYNE2 | Not considered actionable at time of analysis |  |
| TBC1D4 | Not considered actionable at time of analysis |  |
| TET2 | Mutations | "High risk" factor of myeoldysplastic or myeloproliferative disorders required for trial enrollment. |
| TGFb1 | Not considered actionable at time of analysis |  |
| TGFBR2 | Not considered actionable at time of analysis |  |
| TNFAIP3 | Not considered actionable at time of analysis |  |
| TOP1 | Not considered actionable at time of analysis |  |
| TOP2A | Copy number changes | Treatment with topoisomerase 2A inhbitors |
| TP53 | Not considered actionable at time of analysis |  |
| TSC1 | Inactivating mutations or deletions | Treatment with mTOR inhibitors |
| TSC2 | Inactivating mutations or deletions | Treatment with mTOR inhibitors |
| TSHR | Not considered actionable at time of analysis |  |
| USH2A | Not considered actionable at time of analysis |  |
| VHL | Not considered actionable at time of analysis |  |
| WHSC1 | Not considered actionable at time of analysis |  |
| WT1 | Not considered actionable at time of analysis |  |
| ZNF238 | Not considered actionable at time of analysis |  |
| ZNF536 | Not considered actionable at time of analysis |  |

| **eTable 2.** List of concordance between the CLIA and research platform | | | |
| --- | --- | --- | --- |
| **Gene** | **Number of mutations detected in CLIA hotspot limited gene panel** | **Number of mutations detected in CLIA hotspot limited gene panel and in deep targeted sequencing platform (T200)** | **Number of mutations detected in CLIA hotspot limited gene panel not detected in deep targeted sequencing platform (T200)** |
| ABL1 | 2 | 1 | 1 |
| AKT1 | 10 | 10 | 0 |
| ALK | 1 | 1 | 0 |
| ATM | 9 | 9 | 0 |
| BRAF | 108 | 106 * | 1 |
| CDKN2A | 12 | 10 * | 1 |
| CSF1R | 4 | 4 | 0 |
| EGFR | 17 | 16 * | 0 |
| ERBB2 | 5 | 5 | 0 |
| ERBB4 | 6 | 6 | 0 |
| FGFR1 | 4 | 4 | 0 |
| FGFR2 | 8 | 8 | 0 |
| FGFR3 | 5 | 5 | 0 |
| FLT3 | 3 | 3 | 0 |
| GNA11 | 1 | 1 | 0 |
| HRAS | 8 | 7 | 1 |
| IDH1 | 74 | 74 | 0 |
| IDH2 | 5 | 4 * | 0 |
| JAK3 | 1 | 1 | 0 |
| KDR | 11 | 11 | 0 |
| KIT | 18 | 16 * ¶ | 0 |
| KRAS | 124 | 120 * | 1 |
| MET | 6 | 6 | 0 |
| NRAS | 51 | 51 | 0 |
| PDGFRA | 4 | 4 | 0 |
| PIK3CA | 134 | 132 * | 0 |
| PTEN | 18 | 14 * ¶ | 1 |
| RET | 9 | 9 | 0 |
| SMO | 3 | 3 | 0 |
| STK11 | 2 | 2 | 0 |
| *12 mutations found on research platform but below reporting threshold. ¶ 2 indels not being reported on the research platform at the time the informatics analysis was done | | | |

| **eTable 3. Alterations sent for validation** | | | | | |
| --- | --- | --- | --- | --- | --- |
| **Gene** | **Test ordered** | **Did validation confirm finding?** | **Alteration** | **MAF** | **Copy Number** |
| AKT1 | Baylor Microarray Analysis Test Code 9510 | No | Amplification | n/a | 4.3 |
| AKT1 | Baylor Microarray Analysis Test Code 9511 | No | Amplification | n/a | 4.7 |
| ALK | Baylor College of Medicine Test code 9140 | Yes | Mutation | 30% | n/a |
| ATM | Baylor College of Medicine Test code 9141 | Yes | Mutation | 39% | n/a |
| CDK4 | Baylor Microarray Analysis Test Code 9515 | Yes | Amplification | n/a | 4.5 |
| CDK4 | Baylor Microarray Analysis Test Code 9511 | Yes | Amplification | n/a | 20.5 |
| CDK4 | 9510 400K cGH/SNP Array | Yes | Amplification | n/a | 26.8 |
| CDKN2A | Baylor College of Medicine Test code 9140 | Yes | Mutation | 16% | n/a |
| CDKN2A | Baylor College of Medicine Test code 9140 | Yes | Mutation | 31% | n/a |
| EGFR | Baylor College of Medicine Test code 9141 | No | Mutation | 9% | n/a |
| EGFR | EGFR IHC | Yes | Amplification | n/a | 5.6 |
| EGFR | EGFR FISH | Yes | Amplification | n/a | 6.9 |
| EGFR | EGFR IHC | Yes | Amplification | n/a | 12.1 |
| EGFR | Foundation One | Yes | Amplification | n/a | 13.8 |
| EGFR | EGFR FISH | Yes | Amplification | n/a | 21.1 |
| EGFR | EGFR FISH | Yes | Amplification | n/a | 25.7 |
| EGFR | Baylor Microarray Analysis Test Code 9515 | Yes | Amplification | n/a | 31.6 |
| EGFR | EGFR IHC | Yes | Amplification | n/a | 32.7 |
| EGFR | EGFR FISH | Yes | Amplification | n/a | 41.8 |
| EGFR | EGFR FISH | yes | Amplification | n/a | 53 |
| ERBB2 | HER2/neu FISH | No | Amplification | n/a | 3.1 |
| **Gene** | **Test ordered** | **Did validation confirm finding?** | **Alteration** | **MAF** | **Copy Number** |
| ERBB2 | HER2/neu FISH | No | Amplification | n/a | 4 |
| ERBB2 | HER2/neu FISH | No | Amplification | n/a | 4.1 |
| ERBB2 | HER2/neu FISH | No | Amplification | n/a | 4.2 |
| ERBB2 | HER2/neu FISH | No | Amplification | n/a | 4.4 |
| ERBB2 | HER2/neu FISH | No | Amplification | n/a | 4.4 |
| ERBB2 | HER2/neu FISH | No | Amplification | n/a | 4.4 |
| ERBB2 | HER2/neu FISH | No | Amplification | n/a | 4.6 |
| ERBB2 | HER2/neu FISH | No | Amplification | n/a | 5.3 |
| ERBB2 | HER2/neu FISH | No | Amplification | n/a | 5.6 |
| ERBB2 | HER2/neu FISH | No | Amplification | n/a | 6.8 |
| ERBB2 | HER2/neu FISH | Yes | Amplification | n/a | 4.5 |
| ERBB2 | IHC | Yes | Amplification | n/a | 4.5 |
| ERBB2 | Foundation One | Yes | Amplification | n/a | 8.3 |
| ERBB2 | HER2/neu FISH | Yes | Amplification | n/a | 26.9 |
| FGFR1 | FGFR1 FISH | Yes | Amplification | n/a | 8.8 |
| FGFR1 | Knight Diagnostic Lab | Yes | Amplification | n/a | 12.4 |
| FGFR1 | FGFR1 FISH | Yes | Amplification | n/a | 12.6 |
| FGFR1 | Foundation One Testing | Yes | Amplification | n/a | 13 |
| FGFR1 | Knight Diagnostic Lab | Yes | Mutation | 12% | n/a |
| FGFR3 | ARUP MIP Array | No | Amplification | n/a | 5 |
| FGFR3 | Baylor Microarray Analysis Test Code 9515 | No | Amplification | n/a | 5.7 |
| FGFR3 | Baylor Microarray Analysis Test Code 9515 | No | Amplification | n/a | 7.1 |
| FGFR3 | Baylor College of Medicine Test code 9140 | No | Mutation | 8% | n/a |
| Met | c-Met FISH | No | Amplification | n/a | 3,5.3 |
| Met | c-Met FISH | No | Amplification | n/a | 3.5,6.7 |
| Met | c-Met FISH | No * | Amplification | n/a | 4.1 |
| Met | c-Met FISH | Yes | Amplification | n/a | 8.1 |
| **Gene** | **Test ordered** | **Did validation confirm finding?** | **Alteration** | **MAF** | **Copy Number** |
| Met | c-Met FISH | Yes | Amplification | n/a | 16.8 |
| MTOR | Baylor College of Medicine Test code 9140 | yes | Mutation | 30% | n/a |
| NF1 | Baylor College of Medicine Test code 9140 | No | Mutation | 8% | n/a |
| PIK3R1 | Baylor College of Medicine Test code 9140 | No | Mutation | 14% | n/a |
| PIK3R1 | Baylor College of Medicine Test code 9140 | Yes | Mutation | 32% | n/a |
| PIK3R1 | Baylor College of Medicine Test code 9140 | Yes | Mutation | 4% | n/a |
| PTEN | PTEN IHC | No | Deletion | n/a | 0.3 |
| PTEN | PTEN IHC | No | Deletion | n/a | 0.7,0.1 |
| PTEN | PTEN IHC | No | Deletion | n/a | 0.8, 0.4 |
| PTEN | PTEN IHC | No | Deletion | n/a | 1.0, 0.7 |
| PTEN | PTEN IHC | Yes | Deletion | n/a | 0.8 |
| PTEN | PTEN IHC | Yes | Deletion | n/a | 0.9 |
| PTEN | Baylor College of Medicine Test code 9140 | Yes | Mutation | 33% | n/a |
| PTEN | Baylor College of Medicine Test code 9140 | Yes | Mutation | 50% | n/a |
| PTEN | Baylor College of Medicine Test code 9140 | Yes | Mutation | 12% | n/a |
| PTEN | Baylor College of Medicine Test code 9140 | Yes | Mutation | 81% | n/a |
| FISH :Fluorescence in situ hybridization, IHC:immunohistochemistry, MAF= Mutant allelic frequency, N/A: Not Applicable, | | | | | |

* denotes conflicting result of patient that tested positive in CLIA immunohistochemistry (IHC) but negative in Fluorescence in situ hybridization (FISH)
